# Serotypes *of Streptococcus suis* isolated from healthy pigs in Phayao Province, Thailand

**DOI:** 10.1186/s13104-016-2354-2

**Published:** 2017-01-19

**Authors:** P. Thongkamkoon, T. Kiatyingangsulee, M. Gottschalk

**Affiliations:** 1 Veterinary Research and Development Center (Upper Northern Region), 221 M.6, Wiengtarn, Hangchat, Lampang, 52190 Thailand; 2 Department of Livestock Development, National Institute of Animal Health, Chatuchak, Bangkok, 10900 Thailand; 3 Faculty of Veterinary Medicine, University of Montreal, 3200 Sicotte, Saint-Hyacinthe, QC J2S 2M2 Canada

**Keywords:** *Streptococcus suis*, Serotypes, Tonsil, Pigs, Phayao Province

## Abstract

**Background:**

*Streptococcus suis* (*S. suis*) is an important swine and human pathogen. There are 33 serotypes that have been described. Zoonotic cases are very common the Northern part of Thailand, especially in Phayao Province. However, the prevalence of *S. suis* and, more particularly the different serotypes, in pigs in this region is poorly known and needed to be addressed.

**The context and purpose of the study:**

Distribution of *S. suis* serotypes varies depending on the geographical area. Knowledge of the serotype distribution is important for epidemiological studies. Consequently, 180 tonsil samples from slaughterhouse pigs in Phayao Province had been collected for surveillance, from which 196 *S. suis* isolates were recovered. Each isolate was subcultured and its serotype identified using multiplex PCR. Slide agglutination combined with precipitation tests were used following multiplex PCR to differentiate the isolates showing similar sizes of amplified products specific to either serotype 1 or 14 and 2 or 1/2. Non-typable isolates by multiplex PCR were serotyped by the coagglutination test.

**Results:**

Of the 196 isolates, 123 (62.8%) were typable and 73 (37.2%) were non-typable. This study revealed the presence of serotypes 1, 1/2, 2, 3, 4, 5, 7, 9, 11, 12, 13, 14, 21, 22, 23, 24, 25, 29, and 30. Serotype 23 was the most prevalent (20/196, 10.2%), followed by serotype 9 (16/196, 8.2%), serotype 7 (16/196, 8.2%), and serotype 2 (11/196, 5.6%). The latter is the serotype responsible for most human cases.

**Conclusion:**

Almost all serotypes previously described are present in Northern Thailand. Therefore, this report provides useful data for future bacteriological studies.

## Background

*Streptococcus suis* (*S. suis*) is a pig pathogen that can cause severe diseases in humans. Most infections in humans are the result of exposure to sick or carrier pigs, including raw pork contaminated by *S. suis* [1, 32]. The main route of infection in humans is by penetration through injured skin on the hands and/or arms after contact with contaminated animals, carcasses or meat [35]. This organism naturally inhabits the nasal cavities and tonsils of healthy pigs, which act as a reservoir or carrier [11, 36]. Weaning pigs and piglets are more susceptible to infection [33].

To date, *S. suis* has been classified into 33 serotypes (1–31, 33, and 1/2) [15]. Serotype of *S. suis* may vary depending on the geographical area. The most prevalent *S. suis* in slaughterhouse pigs in southern Vietnam is serotype 2 [12], whereas the predominant serotypes in South Korea are serotypes 3 and 4 [17]. There is variation in virulence among serotypes. Serotypes 2 and 1/2 are the most virulent and the most frequently isolated from diseased animals in North America [7]. Alongside, serotype 2 is highly virulent in humans [39]. Serotypes 4, 5, 14, 16, 21, and 24 have also been found in limited human cases [10, 23]. However, isolation of a specific serotype from carrier pigs is difficult because low-pathogenic serotypes and non-typable strains compete for the same target site in the tonsils [5].

In Thailand, *S. suis* has been a major public health problem, especially in the Northern and Northeastern regions [16]. The main route of infection in humans in these areas is the oral route due to particular ethnic behaviors where consumption of raw pork and meat products occurs [3]. In contrast, most patients acquire the disease following occupational exposure to pigs or pork products in Western countries [4]. Human cases of *S. suis* infections in humans in Thailand have been reported since 1987 [26]. In 2010, there was an outbreak of *S. suis* in the Northern part of Thailand. The Ministry of Public Health reported 171 human cases during this outbreak. In Phayao, a province in the Northern part of Thailand also highly affected by *S. suis*, there was 1 fatal case and 25 non-fatal cases of infection reported, with signs of deafness (following meningitis) and/or arthritis [20]. However, there is a lack of epidemiological data which associated swine with human cases. Therefore, the survey of *S. suis* in tonsils of slaughtered pigs in Phayao Province was carried out [22]. The objective of this study was to identify and provide serotype data of *S. suis* isolates from a previous study which will be useful for bacteriological studies.

## Methods

### Bacterial strains and isolates

*Streptococcus suis* serotype 1, 2, 7, and 8 strains (NCTC10237, NCTC10234, NCTC10155, NCTC10156) were obtained from the National Institute of Animal Health, Japan. *S. suis* serotype 1/2 (2651), 9 (DAN22083), and 14 (DAN13730) were obtained from the Faculty of Veterinary Medicine, University of Montreal, Canada. These strains were used as the positive controls for multiplex PCR, slide agglutination test, and precipitation test.

One hundred and ninety-six *S. suis* were isolated from the palatine tonsils of 180 apparently clinically healthy pigs aged 5–6 month and weighing 90–100 kg, and intended for human consumption. Ten pigs belonging to various farms were randomly sampled from each of 18 local slaughterhouses in Phayao Province, Northern part of Thailand, during January to March 2010. Consent was obtained from slaughterhouse owners to collect all of the samples from carcasses. In summary, the pig samples belonged to 46 small holding farms located in Phayao Province: 6.5% of farms (3/46) and 16.1% of pigs (29/180) were negative for *S. suis*. The isolates were stored in a −80 °C germbank, Bacteriology section, National Institute of Animal Health. These samples were exempt from an ethics committee or IBR of the National Institute of Animal Health (NIAH) Thailand due to the ethics committee being founded the year after samples were collected. Furthermore, all samples were collected from carcasses, whereas the ethics committee concerns only alive animals, as indicated by the definition of “animals” in “the animals for scientific purposes act, B.E. 2558 (A.D. 2015), Thailand”.

### Bacterial subculture

*Streptococcus suis* isolates from the germbank were subcultured on 5–7% sheep blood agar (Oxoid^®^, UK)and incubated at 37 °C with 5% carbon dioxide for 24 h. Colony characterization and biochemical testing were done to verify that the agent was *S. suis* [27]. Afterwards, each isolate was further identified with the API^®^ 20 strep test kit (bioMérieux, USA).

### *Streptococcus suis* confirmation and serotype identification

**Fig. 1:**
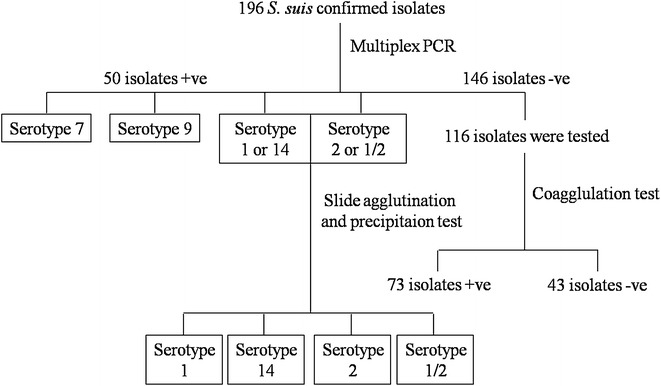
Flow chart showing serotype identification steps of *S. suis* isolates

*Streptococcus suis* confirmation was performed by PCR modified from Marois et al. [18] and serotyping was conducted using the following three techniques: multiplex PCR, slide agglutination combined with precipitation test, and the coagglutination test, as described in Fig. 1.

#### Multiplex PCR

Firstly, all 196 *S. suis* isolates were confirmed and tested for serotypes 1, 1/2, 2, 7, 9, and 14 as well as for *S. suis* species by multiplex PCR. Briefly, DNA of each isolate and the reference strains were prepared using a boiling method and subsequently reacted with the primers specific for the 16S rRNA and capsular genes of *S. suis* serotypes 1, 2, 7, and 9 [29, 30, 38].

#### Slide agglutination combined with precipitation test

**Fig. 2:**
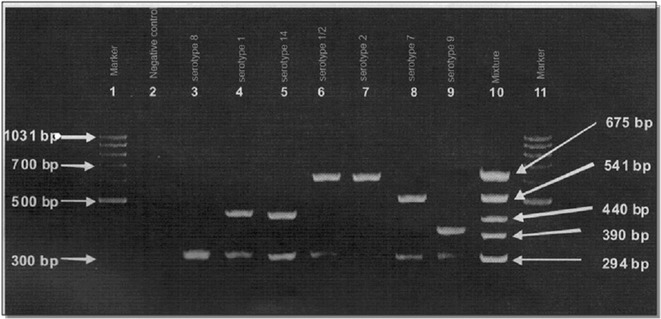
Amplification of multiplex PCR of reference *S. suis*. *Lane 1* and *11*, DNA marker (Fermentas, USA); *Lane 2*, Negative control (DW); *Lane 3*, serotype 8; *Lane 4*, serotype 1; *Lane 5*, serotype 14; *Lane 6*, serotype1/2; *Lane 7*, serotype 2; *Lane 8*, serotype 7; *Lane 9*, serotype 9; *Lane 10*, mixture of serotypes 1, 2, 7, 9, and 14

The amplified products of *S. suis* serotypes 1 versus 14 and 2 versus 1/2 showed similar sizes by multiplex PCR (Fig. 2). Therefore, they were subsequently differentiated by slide agglutination combined with precipitation test. The antisera for testing serotype 1 and 2 were prepared in NIAH, Thailand [25], while the antiserum for serotype 14 was obtained from the Staten Serum Institute, Copenhagen, Denmark. Slide agglutination test was performed by mixing 1–2 colonies of *S. suis* with a drop of specific antiserum on a slide. Agglutination between the homologous antigen and antiserum should be observed. The precipitation test was performed in capillary tubes as described elsewhere [28]. Briefly, the microhematocrit tube (Vitrex Medical, Denmark) was dipped into the tube containing specific antiserum until the antisera filled up to 1 cm. Subsequently, the same volume of extracted antigen of the suspected *S. suis* serotype was also filled and the precipitation line was observed within 15 min.

#### Coagglutination test

Finally, 146 *S. suis* isolates unidentifiable by the former methods were subcultured on blood agar plates for 24 h, after which 1 colony was inoculated into 10 ml of Todd Hewitt broth and incubated at 37 °C for 24 h. The growth culture of the isolate required an optical density of 0.6 or higher at a wavelength of 600 nm, or was otherwise re-incubated for a few hours. Bacteria were then harvested by centrifugation, resuspended in 3 ml of 0.2% formalin in phosphate buffered saline, and killed bacteria kept at 4 °C. Of the 146 isolates, 30 isolates were excluded from the test because cultures did not reach the optimal growth. Therefore, 116 killed *S. suis* isolates were sent to the Faculty of Veterinary Medicine, University of Montreal for serotyping using the polyvalent and monovalent coagglutination test with antisera against all 33 serotypes of *S. suis* [8].

## Results

By multiplex PCR and slide agglutination combined with precipitation test, 6 serotypes were identified. The number of the isolates identified as serotype 1, 1/2, 2, 7, 9, and 14 were 2, 10, 10, 16, 9, and 3 isolates, respectively. Many of these (146/196) were negative. The 116 unidentified serotype isolates were further tested by coagglutination test. This test revealed most serotypes, including 2, 3, 4, 5, 9, 11, 12, 13, 21, 22, 23, 24, 25, 29, and 30 to which belonged 73 isolates, while the serotypes of 43 isolates could not be identified. In summary, serotyping of 196 *S. suis* isolates using 3 methods identified 123 (62.8%) isolates as serotypes 1, 1/2, 2, 3, 4, 5, 7, 9, 11, 12, 13, 14, 21, 22, 23, 24, 25, 29, and 30, while 73 (37.2%) isolates remained non-typable (Table 1). The distribution of *S. suis* serotypes among districts in Phayao province was scattered (Table 2).

**Table 1:** The results of serotype identification by multiplex PCR, slide agglutination combined with precipitation test, and coagglutination test

Serotype test^a^	1	1/2	2	3	4	5	7	9	11	12	13	14	21	22	23	24	25	29	30	Non-typable	Number of isolates tested
1 and 2	2	10	10	ND	ND	ND	16	9	ND	ND	ND	3	ND	ND	ND	ND	ND	ND	ND	146	196
3	0	0	1	10	6	5	0	7	1	1	2	0	4	1	20	8	1	2	4	43	116
Total (%)	2 (1.0)	10 (5.1)	11 (5.6)	10 (5.1)	6 (3.1)	5 (2.6)	16 (8.2)	16 (8.2)	1 (0.5)	1 (0.5)	2 (1.0)	3 (1.5)	4 (2.0)	1 (0.5)	20 (10.2)	8 (4.1)	1 (0.5)	2 (1.0)	4 (2.0)	73 (37.2)	196 (100)

*ND* not done

^a^1 = multiplex PCR, 2 = slide agglutination combined with precipitation test, 3 = coagglutination test

**Table 2:** Distribution of the serotypes of *Streptococcus suis* isolates in different districts of Phayao Province

District of Phayao Province	Number of isolates																			Total
	Serotype																			
	1	1/2	2	3	4	5	7	9	11	12	13	14	21	22	23	24	25	29	30	
Dok kham tai	1	3	2	1	1	2	3	4				1			2					20
Phu kam yao				2			1	1					1		2					7
Muang Phayao	1	6	5	4	3	2	7	5	1	1	1	2	2	1	9	1	1		1	53
Chun				1									1			1			2	5
Mae chai			1	1			4	6			1				2	1		1		17
Pong			1		1		1								2	1				6
Chaing kham				1											3	1		1	1	7
Phu sang		1	2		1											1				5
Chuang muan						1										2				3
Total	2	10	11	10	6	5	16	16	1	1	2	3	4	1	20	8	2	2	4	123

## Discussion

A total of 196 *S. suis* isolates were studied, which consisted of 123 typable (62.8%) and 73 non-typable isolates (37.2%). The typable and non-typable isolates were widely distributed within the province regardless of farms and slaughterhouse location. However, the 73 non-typable isolates included 6 isolates that reacted with multiple serotypes: a first isolate with serotypes 4 and 9, a second isolate with serotypes 6, 12, 23, and 30, and a third isolate with serotypes 6, 23, and 30, while three more isolates reacted with serotypes 23 and 30. Isolates reacting with multiple serotypes are commonly isolated from tonsils [2]. Multiple serotypes may be the result of similar capsular polysaccharide composition among the different serotypes, which are probably due to recombination [13]. Non-typable isolates were usually isolated from diseased pigs with different proportions that varied from 12 to 26% [19]. Our results, where a higher proportion was obtained, might be due to the fact that the isolates were from tonsils and not from diseased animals, as was previously described. Moreover, some isolates that did not react with any specific antisera might either be truly novel serotypes or previously described but non-encapsulated isolates [10]. To date, all 35 serotypes of *S. suis* could be differentiated by newly developed multiplex PCR assays, and it was found that more than 42% of non-encapsulated non-typable strains (using the coagglutination test) were typable by this method [24]. Therefore, we will apply the new multiplex PCR to further studies.

Most of the isolates we found were serotype 23 (20, 10.2%) followed by serotype 9 (16, 8.2%), serotype 7 (16, 8.2%), and serotype 2 (11, 5.6%). This differs from strains of *S. suis* collected from slaughterhouses in Vietnam, in which serotype 2 (45/317, 14.2%) was the most common [12]. Even though Thailand and Vietnam are located in the same region, South-East Asia, and samples were collected during the same period, the prevalence of *S. suis* serotypes is different.

Serotypes 1–9, 1/2, and 14, which cause disease in pigs [4, 37] were also found in this study, except for serotype 8, which reflects the high risk of streptococcosis in pigs in Phayao Province. On the other hand, while reports frequently found serotypes 17, 18, 19, and 21 in healthy pigs [9, 14, 21], we observed only serotype 21 (4/123, 3.3%). Nevertheless, serotype 21 was confirmed as a cause of meningitis in one human case [10]. Therefore, healthy carrier pigs are still considered as potential health hazards for individuals working with or in close contact with pigs [11, 34].

Serotype 2, the most virulent and most frequently associated with disease in both pigs and humans [6, 35], was attributed to 11 of the 196 strains (5.6%), which was rather low. On the other hand, serotype 2 human cases reported in Thailand are relatively high [31], possibly due to the fact that pork from one clinically diseased pig was distributed to a group of people. As reported by the Ministry of Public Health, almost all human patients had eaten improperly cooked pork from the same funeral ceremony. As we mentioned before, certain ethnic and cultural behaviors, such as consumption of raw blood and meat, are the main cause of disease in Thailand.

## Conclusion

This study reports many of the known serotypes in pigs in Thailand rather than only some serotypes, as was previously observed in other studies, which were limited due to specific capsular antisera for the coagglutination test. Therefore this study provides useful data for future bacteriological studies.

## Abbreviations

*S. suis*: *Streptococcus suis*
DW: distilled water
PCR: polymerase chain reaction
cm: centimeter
nm: nanometer
ml: millimeter
°C: degree celsius
